# Safety Profile of Ibrutinib: An Analysis of the WHO Pharmacovigilance Database

**DOI:** 10.3389/fphar.2021.769315

**Published:** 2021-10-28

**Authors:** Marion Allouchery, Cécile Tomowiak, Thomas Lombard, Marie-Christine Pérault-Pochat, Francesco Salvo

**Affiliations:** ^1^ Pharmacologie Clinique et Vigilances, CHU de Poitiers, Poitiers, France; ^2^ Faculté de Médecine, Université de Poitiers, Poitiers, France; ^3^ Université de Bordeaux, INSERM, BPH, UMR1219, Bordeaux, France; ^4^ Onco-Hématologie et Thérapie Cellulaire, CHU de Poitiers, Poitiers, France; ^5^ INSERM CIC 1402, CHU de Poitiers, Poitiers, France; ^6^ Pharmacie à Usage Intérieur, CHU de Poitiers, Poitiers, France; ^7^ Laboratoire de Neurosciences Expérimentales et Cliniques, INSERM, UMR1084, Université de Poitiers, Poitiers, France; ^8^ CHU de Bordeaux, Pôle de Santé Publique, Service de Pharmacologie Médicale, Bordeaux, France

**Keywords:** ibrutinib, Bruton’s tyrosine kinase inhibitor, drug safety, adverse drug reaction, pharmacovigilance

## Abstract

As ibrutinib has become a standard of care in B-cell malignancies in monotherapy or in combination with other agents, definition of its safety profile appears essential. The aim of this study was to further characterize the safety profile of ibrutinib through the identification of potential safety signals in a large-scale pharmacovigilance database. All serious individual case safety reports (ICSRs) in patients aged ≥18 years involving ibrutinib suspected in the occurrence of serious adverse drug reactions or drug interacting from November 13th, 2013 to December 31st, 2020 were extracted from VigiBase, the World Health Organization global safety database. Disproportionality reporting was assessed using the information component (IC) and the proportional reporting ratio (PRR), with all other anticancer drugs used as the reference group. To mitigate the confounding of age, two subgroups were considered: patients aged<75 years and ≥75 years. A signal of disproportionate reporting (SDR) was defined if both IC and PRR were significant. A total of 16,196 ICSRs were included. The median age of patients was 72.9 years, 42.6% of ICSRs concerned patients aged ≥75 years, and 64.2% male patients. More than half (56.2%) of ICSRs resulted in hospitalization or prolonged hospitalization. Among 713 SDRs, 36 potential safety signals emerged in ibrutinib-treated patients, mainly ischemic heart diseases, pericarditis, uveitis, retinal disorders and fractures. All potential safety signals having arisen in this analysis may support patient care and monitoring of ongoing clinical trials. However, owing to the mandatory limitations of this study, our results need further confirmation using population-based studies.

## Introduction

Ibrutinib, a first-in-class, oral, once-daily, Bruton’s tyrosine kinase (BTK) inhibitor, has been demonstrated as an effective treatment for chronic lymphocytic leukemia (CLL), mantle cell lymphoma (MCL), Waldenström macroglobulinemia (WM), marginal zone lymphoma and chronic graft versus host disease (Treon et al., 2015; Chanan-Khan et al., 2016; Miklos et al., 2017; Dimopoulos et al., 2018; Burger et al., 2019; Byrd et al., 2019; Moreno et al., 2019; Shanafelt et al., 2019). By targeting BTK, ibrutinib impairs B-cell receptor (BCR) signaling pathway and inhibits B-cell proliferation, survival and migration, leading to significant prolonged survival in high-risk, relapsed or refractory diseases (Herman et al., 2011; Ponader et al., 2012).

Discrepancies in discontinuation rates due to toxicity have been highlighted between initial pivotal clinical trials and real-world studies, where adverse drug reactions (ADRs) were responsible for 51, 29, and 21% of ibrutinib discontinuations in CLL, WM and MCL respectively (Gustine et al., 2018; Mato et al., 2018; Sharman et al., 2020). Higher treatment discontinuation for safety reasons in real-world settings is likely due to differences in patient characteristics. Ibrutinib is prescribed mostly to elderly patients for whom chemo-immunotherapy is unsuitable. As a result, comorbidity burden and co-medications could compromise the safety of ibrutinib in real-life practice. In a previously published cohort study (*n* = 102 patients), patients aged ≥80 years were at higher risk of serious adverse drug reaction (SADR) within the first year of ibrutinib treatment (Allouchery et al., 2021).

Despite increasing use in B-cell malignancies, the post-marketing safety profile of ibrutinib remains unclear. While several studies have assessed the safety of ibrutinib in real life settings, but they have been focused on specific ADRs, such as infectious, bleeding or cardiovascular ADRs (Lipsky et al., 2015; Varughese et al., 2018; Dartigeas et al., 2019; Dickerson et al., 2019; Salem et al., 2019; Frei et al., 2020). Unlike chemotherapy, which is given for a finite number of cycles, ibrutinib is continued until the occurrence of disease progression or unacceptable toxicity. Data on long-term safety of ibrutinib are especially important in light of prolonged ibrutinib exposure but are mainly limited to clinical trial settings. In the 6-years follow-up in the phase 3 RESONATE study of relapsed/refractory CLL (*n* = 195 patients, median ibrutinib treatment duration of 41 months), the most commonly treatment-emergent adverse events of any grade remained consistent with previous reports of patients treated with ibrutinib, and generally decreased over time for patients remaining on ibrutinib therapy, with exceptions for hypertension and bleeding ADRs (Munir et al., 2019).

As ibrutinib has become a standard of care in B-cell malignancies in monotherapy or in combination with other agents, definition of its safety profile appears essential. The aim of this study was to further characterize the safety profile of ibrutinib through identification of potential safety signals in a large-scale pharmacovigilance database.

## Materials and Methods

### Data Source

The Uppsala Monitoring Center (UMC) receives individual case safety reports (ICSRs) of suspected ADRs from national pharmacovigilance systems, which are stored in VigiBase, the World Health Organization (WHO) global database of ICSRs (Lindquist, 2008). In December 2020, VigiBase contained more than 24 million ICSRs from >130 countries. ADRs originate from physicians, pharmacists or other healthcare professionals, patients and pharmaceutical companies.

Each ICSR contains 1) anonymous administrative informations (reporter qualification, date of reporting, country of origin); 2) patient characteristics (sex, age); 3) description of the ADRs coded according to the Medical Dictionary for Regulatory Activities (MedDRA) (Brown et al., 1999), seriousness, time to onset, outcome; 4) drugs involved: international nonproprietary name and coded according to the Anatomical Therapeutic Chemical (ATC) classification, role in the ADRs, indication.

Per the Jardé law in France regarding research involving human participants, this study did not require ethical review or informed consent as it involved an existing anonymized database.

### Data Extraction and Selection

All serious ICSRs involving ibrutinib (ATC code: L01XE27) suspected in the occurrence of SADRs or drug interacting from November 13th, 2013 (first authorization in the United States) to December 31st, 2020 were extracted. Exclusion criteria were as follows: missing age, patient aged <18 years, no MedDRA preferred term (PT) and PT “no adverse event.”

### Data Analysis

A descriptive analysis of included ICSRs was performed. Continuous variables were described by mean and standard deviation, or median and interquartile range (IQR) and categorical variables by number and proportion of subjects in each class.

Disproportionality analyses of spontaneous reporting databases are based on the identification of drug-event pairs reported more often than expected regarding the frequency of reporting of other drug-event pairs, resulting in signals of disproportionate reporting (SDRs) (Montastruc et al., 2011). In the present study, SDR detection was performed using the information component (IC) with its 95% credibility interval (IC_025_) and the proportional reporting ratio (PRR) with its corresponding 95% confidence interval (95%CI). Specifically developed and validated by UMC, the IC is an indicator value for disproportionate reporting that compares observed and expected values, the objective being to find associations between drugs and ADRs (Bate et al., 1998). The PRR compares the rate of reporting of an event among all reports for a given drug with the rate of reporting of the same event among a control group of drugs (Evans et al., 2001). The IC and the PRR were calculated for ibrutinib in comparison with all other anticancer drugs (ATC code: L01 “antineoplastic agents”), considering SADRs at the PT level. All other anticancer drugs were used as comparators because 1) the choice of relevant comparators for ibrutinib is complex since ibrutinib is mostly prescribed in elderly patients for whom chemo-immunotherapy is unsuitable; 2) other anticancer drugs prescribed in the same approved indications as ibrutinib, like rituximab or cyclophosphamide, are also used in a broad spectrum of indications in solid tumors or in hematological malignancies; 3) the large number of ICSRs with all other anticancer drugs allows for sufficient sensitivity to detect ibrutinib-associated safety signals. To mitigate the impact of age on safety profile of ibrutinib, two subgroups were considered: patients aged <75 years and ≥75 years. Drug-SADR associations were statistically significant if IC_025_ ≥ 1, or if the lower limit of 95% confidence interval of the PRR >1, with at least 3 cases of interest reported. A SDR was considered if both measures, i.e., IC and PRR, were significant. SDRs were then assessed carefully by two clinical pharmacologists trained in pharmacovigilance (MA, M-CP-P), according to their clinical relevance and their acknowledgment in the Summary of Product Characteristics (SmPC) approved by the European Medicines Agency (EMA) (European Medicines Agency, 2021) and by the US Food and Drug Administration (FDA) (US Food and Drug Administration, 2020). All SDRs which correspond to unexpected SADRs were classified as potential safety signals. All statistical analyses were performed using SAS software (v9.4, SAS Institute, NC, United States), and disproportionality analysis was performed by the UMC.

## Results

### Population Characteristics

Among 806,474 patients aged ≥18 years experiencing SADRs associated with anticancer drugs, 16,196 were receiving ibrutinib.

The median (interquartile range, IQR) age of patients was 72.9 (65.0–79.1) years, 42.6% of ICSRs concerned patients aged ≥75 years, and 64.2% male patients. More than half (56.2%) of ICSRs resulted in hospitalization or prolonged hospitalization, 16.4% were fatal and 3.8% were life-threatening. Ibrutinib was the only drug reported as suspected in 84.7% of ICSRs. The indication of ibrutinib treatment was available for 91.8% of ICSRs. The most frequently represented were CLL (65.1%), MCL (11.0%) and WM (6.1%). Two thirds (66.9%) of ICSRs were reported for North America and 28.6% for Europe, and 57.4% were reported by healthcare professionals (Table 1).

**TABLE 1:** Characteristics of included individual case safety reports.

	n = 16,196	
Geographic area, n (%)		
North America	10,832	(66.9)
Europe	4,642	(28.6)
Asia	483	(3.0)
Oceania	145	(0.9)
South America	66	(0.4)
Africa	28	(0.2)
Reporter, n (%)		
Healthcare professional	9,302	(57.4)
Non-health care professional	6,838	(42.2)
Missing data	56	(0.4)
Age, years		
Median, interquartile range	72.9	(65.0–79.1)
Min-max	18–99	
Age, years, n (%)		
<75	9,294	(57.4)
≥75	6,902	(42.6)
Sex, n (%)		
Male	10,390	(64.2)
Female	5,640	(34.8)
Missing data	166	(1.0)
Seriousness criterion^a^, n (%)		
Death	2,651	(16.4)
Life-threatening	613	(3.8)
Caused/Prolonged hospitalization	9,099	(56.2)
Persistent or significant disability/incapacity	201	(1.2)
Other medically important condition	8,177	(50.5)
Missing data	6	(0.0)
Suspected drugs, n (%)		
Only ibrutinib	13,720	(84.7)
Ibrutinib +1 other drug	1,623	(10.0)
Ibrutinib + ≥2 other drugs	853	(5.3)
Indication, n (%)		
Chronic lymphocytic leukemia	10,546	(65.1)
Mantle cell lymphoma	1,781	(11.0)
Waldenström macroglobulinemia	983	(6.1)
Graft versus host disease	121	(0.7)
Marginal zone lymphoma	92	(0.6)
Other	1,346	(8.3)
Missing data	1,327	(8.2)

ICSR, individual case safety report; min-max, minimum-maximum.

a ICSRs can have more than one seriousness criterion.

### Disproportionality Analyses

A total of 1,024 SDRs (713 unique drug-event pairs) were reviewed (patients aged <75 years *n* = 605, ≥75 years *n* = 419). The number and proportion of potential safety signals in each group, as well as expected SADRs, non-signals are displayed in Table 2. A total of 50 SDRs (4.9%) and 36 unique drug-event pairs were classified as potential safety signals (Table 3).

**TABLE 2:** Number of assessed signals of disproportional reporting by age group.

Assessed SDRs	<75 years (n = 605)		≥75 years (n = 419)	
	n	%	n	%
Expected^a^	323	53.4	233	55.6
Non-signal^b^	250	41.4	168	40.1
Potential safety signal	32	5.2	18	4.3

SDR, signal of disproportionate reporting.

a Considered well-described in the summary of product characteristics approved by the European Medicines Agency or by the US Food and Drug Administration.

b SDRs with alternative explanations, or potentially related to the characteristics of ibrutinib-treated patients or B cell malignancies.

**TABLE 3:** Potential safety signals identified in VigiBase, according to age groups.

MedDRA SOC/Sub-group SADRs		Only ibrutinib among suspected drugs %	MedDRA PT	<75 years (n = 9,294)				≥75 years (n = 6,902)			
				n	IC/IC_025_	PRR (95%CI)		n	IC/IC_025_	PRR (95%CI)	
Cardiac disorders	Ischemic heart diseases	84.5	Myocardial infarction	100	1.01/0.71	2.05	(1.68–2.50)	77	NS	NS	
			Angina pectoris	26	1.00/0.39	2.07	(1.40–3.06)	8	NS	NS	
			Coronary artery occlusion	11	1.26/0.28	2.61	(1.43–4.77)	11	1.41/0.44	3.16	(1.68–5.95)
			Ischemic cardiomyopathy	4	2.02/0.28	7.13	(2.54–19.97)	2	NS	NA	
	Pericarditis	76.1	Pericardial effusion	81	1.83/1.50	3.75	(3.00–4.68)	53	1.77/1.35	3.96	(2.95–5.30)
			Pericarditis	24	1.63/1.00	3.35	(2.23–5.05)	11	1.71/0.74	4.19	(2.20–8.01)
			Cardiac tamponade	16	2.13/1.34	5.18	(3.12–8.62)	4	NS	3.23	(1.13–9.24)
			Pericardial hemorrhage	14	3.87/3.01	47.40	(23.95–93.82)	9	2.66/1.57	14.52	(6.21–33.96)
	Bradyarrhythmia (including conduction defects and sinus node dysfunctions)	67.3	Sinus bradycardia	11	1.41/0.43	2.94	(1.61–5.38)	5	NS	NS	
			Right bundle branch block	7	2.21/0.95	6.77	(3.11–14.72)	5	1.96/0.44	6.99	(2.54–19.23)
			Sinus node dysfunction	6	2.10/0.72	6.35	(2.75–14.66)	4	NS	NS	
			Atrioventricular block 1st degree	6	2.49/1.12	10.42	(4.41–24.60)	4	NS	5.24	(1.75–15.68)
			Atrioventricular block 2nd degree	4	1.84/0.10	5.64	(2.04–15.65)	5	NS	3.75	(1.45–9.70)
Ear disorders	Hearing impairment	88.5	Deafness	21	1.27/0.59	2.55	(1.65–3.94)	31	1.54/0.99	3.32	(2.27–4.84)
Endocrine disorders	Hypothyroidism	75.0	Thyroid hormones decreased	5	2.37/0.84	9.96	(3.90–25.46)	3	2.10/0.05	15.73	(3.52–70.28)
Eye disorders	Cataract	85.7	Cataract	52	1.13/0.71	2.25	(1.71–2.96)	67	1.21/0.85	2.50	(1.94–3.22)
	Uveitis	91.3	Uveitis	21	1.16/0.48	2.35	(1.52–3.64)	2	NS	NA	
	Glaucoma	80.8	Glaucoma	16	1.64/0.84	3.45	(2.09–5.70)	10	NS	NS	
	Retinal disorders	92.9	Retinal tear	6	1.93/0.55	5.28	(2.30–12.11)	0	NA	NA	
			Vitreous detachment	5	1.71/0.18	4.46	(1.80–11.01)	0	NA	NA	
			Retinal vascular occlusion	4	2.65/0.91	24.63	(7.84–77.32)	0	NA	NA	
Injury, poisoning and procedural complications	Fractures	93.4	Hip fracture	20	0.95/0.25	2.01	(1.29–3.13)	83	1.36/1.03	2.79	(2.23–3.51)
			Spinal fracture	18	1.16/0.41	2.35	(1.47–3.76)	18	NS	NS	
			Upper limb fracture	10	NS	NS		19	0.83/0.11	1.89	(1.18–3.02)
			Foot fracture	17	1.32/0.55	2.67	(1.65–4.33)	5	NS	NS	
			Lower limb fracture	8	NS	NS		14	1.20/0.84	2.58	(1.48–4.48)
			Spinal compression fracture	15	1.31/0.49	2.68	(1.60–4.49)	6	NS	NS	
			Lumbar vertebral fracture	3	NS	NS		12	1.74/0.81	4.27	(2.29–7.93)
			Stress fracture	7	1.79/0.53	4.39	(2.04–9.42)	0	NA	NA	
Metabolism disorders	Hyponatremia	71.2	Hyponatremia	35	NS	NS		51	0.48/0.06	1.43	(1.08–1.90)
			Blood sodium decreased	28	1.67/1.08	3.42	(2.34–5.00)	39	1.62/1.13	3.53	(2.51–4.95)
Psychiatric disorders	Depression	79.6	Depression	66	0.68/0.31	1.62	(1.27–2.07)	51	1.19/0.77	2.47	(1.85–3.30)
			Depressed mood	20	1.02/0.33	2.13	(1.37–3.33)	16	1.27/0.48	2.73	(1.62–4.59)
Respiratory, thoracic and mediastinal disorders	Pleurisy	82.3	Pleural effusion	247	1.63/1.44	3.21	(2.83–3.64)	221	1.48/1.29	3.08	(2.68–3.54)
			Pleurisy	15	1.42/0.60	2.92	(1.47–4.89)	8	1.22/0.06	2.75	(1.32–5.75)
Vascular disorders	Arterial disorders	92.9	Aortic aneurysm	8	1.70/0.53	3.93	(1.93–8.00)	6	NS	NS	

IC, information component; IC_025_, 95% credibility interval; MedDRA, medical dictionary for regulatory activities; NA, not applicable; NS, not significant; PT, preferred term; PRR, proportional reporting ratio; SADR, serious adverse drug reaction; SOC, system organ class; 95%CI, 95% confidence interval.

Considering cardiac disorders, significant disproportionality emerged for ischemic heart diseases and bradyarrhythmias (including conduction defects and sinus node dysfunctions), with some differences between patients aged <75 years and ≥75 years. Only SDRs of coronary artery occlusion and right bundle branch were found in the older group, but with PRRs similar to those of patients aged <75 years. Ibrutinib was also associated with higher reporting of pericarditis (MedDRA PTs pericarditis, pericardial effusion and pericardial hemorrhage) in both age groups, even though IC and PRR reached statistical significance for cardiac tamponade only for patients aged <75 years. Similarly, regarding vascular SADRs, the SDR of aortic aneurysm was found only in the younger patients.

When focusing on respiratory disorders, a SDR of pleurisy was found for ibrutinib in comparison with other anticancer drugs in the two age groups.

A considerable number of eye disorders related to ibrutinib emerged from our analysis. Cataract was over-reported in both age groups, while uveitis, glaucoma, and retinal disorders (i.e. retinal tear, vitreous detachment and retinal vascular occlusion) were over-reported only in patients aged <75 years.

**FIGURE 1:**
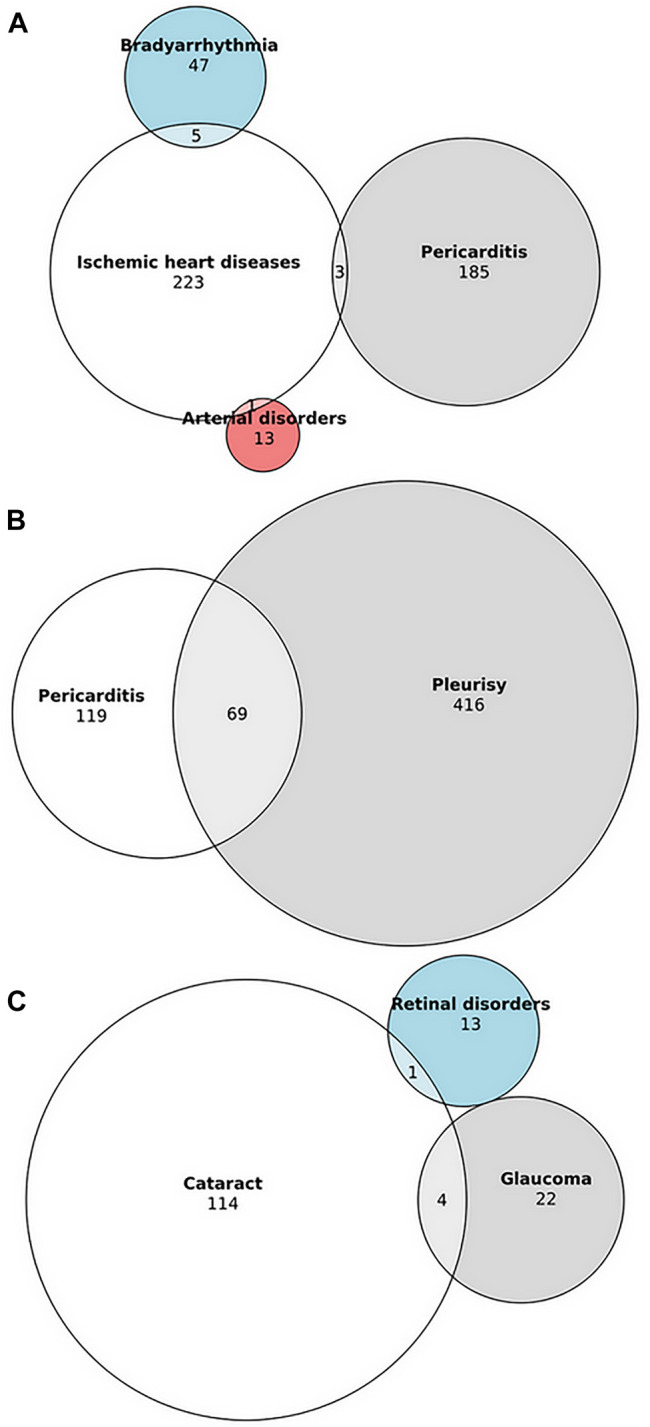
Overlap of cardiovascular, respiratory and ocular serious adverse drug reactions associated with ibrutinib in VigiBase. **(A)** Overlap between ischemic heart diseases, pericarditis, bradyarrhythmia and arterial disorders. **(B)** Overlap between pericarditis and pleurisy. **(C)** Overlap between cataract, glaucoma and retinal disorders. Due to the lack of overlap with other ocular disorders, uveitis is not displayed.

Overlap of cardiovascular, respiratory and ocular SADRs is shown in Figure 1.

We found potential safety signals referred to ibrutinib and fractures. Except for hip fracture, patterns of fractures differ between age groups. Ibrutinib was associated with higher reporting of hip fracture, spinal fracture, foot fracture, spinal compression fracture and stress fracture in patients aged <75 years, and upper and lower limb fractures and lumbar vertebral fracture in patients aged ≥75 years.

Other potential signals of interest were associated with ibrutinib in both age groups, especially deafness, hypothyroidism, hyponatremia and depression.

## Discussion

### Main Results

**FIGURE 2:**
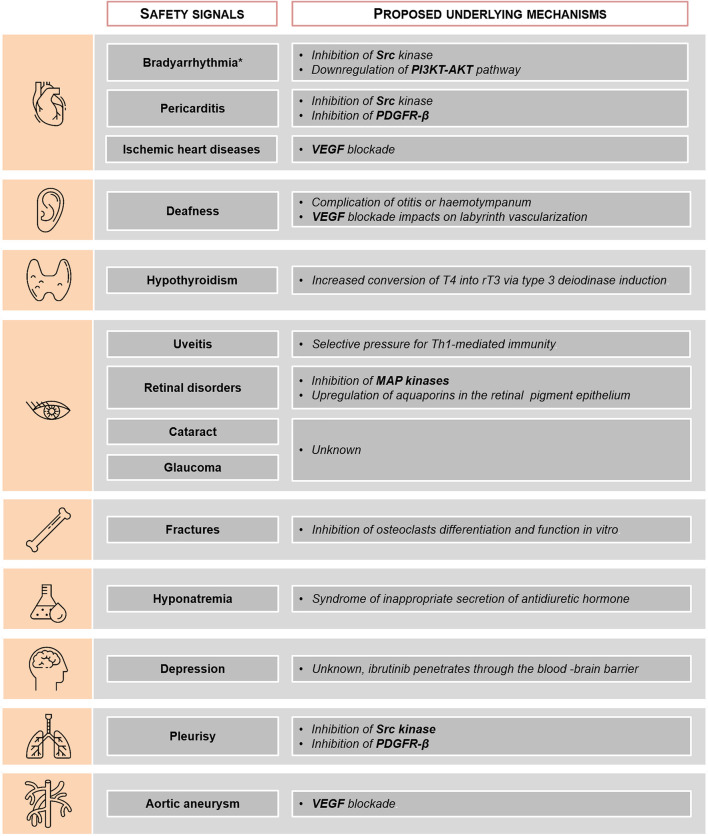
Potential underlying mechanisms for ibrutinib-associated safety signals. *Including conduction defects and sinus node function dysfunctions. PDGFR-β, platelet derived growth factor receptor; MAP, mitogen-activated protein; PI3K, phosphoinositide 3-kinase; rT3, reverse triiodothyronine; T4, thyroxin; VEGF, vascular endothelium growth factor.

We report the largest study to date on the safety profile of ibrutinib through analysis of ICSRs from the WHO pharmacovigilance database. Clinically relevant potential safety signals emerged from our analysis, with some differences between patients aged <75 years and ≥75 years: cardiovascular disorders (including ischemic heart disease, pericarditis, bradyarrhythmia and aortic aneurysm), deafness, hypothyroidism, eye disorders (including cataract, uveitis, glaucoma and retinal disorders), fractures, hyponatremia, depression and pleurisy. Potential underlying mechanisms are summarized in Figure 2.

### Potential Safety Signals

The cardiovascular safety profile of ibrutinib has been widely described, and includes hypertension (Dickerson et al., 2019), supraventricular arrhythmias, including atrial fibrillation (Leong et al., 2016) and life-threatening ventricular arrhythmias (Lampson et al., 2017; Guha et al., 2018). More recently, ibrutinib-associated stroke (with/without AF/hypertension) and cardiac failure have also been listed in the European SmPC. However, our study provides new findings on ibrutinib-associated cardiotoxicity. These results show SDRs of ischemic heart diseases, e.g. myocardial infarction, angina pectoris, coronary artery occlusion and ischemic cardiomyopathy, mostly in ibrutinib-treated patients aged <75 years. While SDRs of myocardial infarction, angina pectoris and ischemic cardiomyopathy disappeared in patients aged ≥75 years, the SDR of coronary artery occlusion still persists in this subgroup, underlining the potential role of ibrutinib. Growing evidence suggest that ibrutinib-associated cardiac toxicity may be explained by the multiple off-target effects of ibrutinib at clinically relevant concentrations. A recent structure-based drug repositioning identified ibrutinib as micromolar Vascular Endothelium Growth Factor Receptor 2 (VEGFR2) inhibitor (Adasme et al., 2020). Despite antiplatelet effects (Busygina et al., 2018, 2019), ibrutinib, like anti-angiogenic drugs, could produce conditions favoring hypertension and ischemic heart diseases, through the inhibition of nitric oxide formation and endothelial dysfunction (Mourad et al., 2008). Vascular Endothelium Growth Factor (VEGF) blockade may also be implicated in arterial wall injuries, leading to increased risk of artery dissections and aneurysms with antiangiogenic drugs (Oshima et al., 2017; Guyon et al., 2021). Interestingly, ibrutinib was shown to interrupt collagen fibrosis in a murine model of chronic graft-versus-host disease (Dubovsky et al., 2014). Furthermore, Wiedower et al. (2016) reported a cerebral aneurysm in a 46-year-old man treated for CLL, with spontaneous resolution after ibrutinib discontinuation, suggesting the possible direct role of ibrutinib on vascular remodeling. In addition to ibrutinib-induced hypertension, VEGF blockade may also explain the SDR of aortic aneurysm with ibrutinib in patients aged <75 years.

Our study identified bradyarrhythmias including conduction defects and sinus node function disorders, as emerging safety signals in patients aged <75 years receiving ibrutinib. In patients aged >75 years, probably due to low number of cases, only a SDR of right bundle branch was found. Conduction disorders signal over-reporting is concordant with a previous pharmacovigilance study in VigiBase evaluating the cardiovascular safety of ibrutinib (Salem et al., 2019). In a recent case-series of ibrutinib-induced high-grade heart block, 14 out of 18 cases of high degree heart block occurred within 13 months of ibrutinib initiation (Vartanov et al., 2021). All patients required pacemaker placement, and most resumed ibrutinib without recurrence of conduction disorders. It remains unclear whether or not the mechanisms that mediate ibrutinib-induced AF may be responsible for conduction disorders. Increased incidence of AF in ibrutinib-treated patients has been associated with not only with inhibition of C-terminal Src kinase (Xiao et al., 2020), but also with downregulation of phosphoinositide 3-kinase (PI3K)-Akt pathway (McMullen et al., 2014; Jiang et al., 2019). Cardiac fibrosis observed in these experimental murine models could contribute to the promotion of conduction disorders.

An association between pericardial/pleural effusions and ibrutinib has been reported in a few case-reports (Styskel et al., 2019; Miatech et al., 2020; Kidoguchi et al., 2021). This is consistent with emerging potential safety signals of pericarditis/pleurisy in both subgroups. A SDR of pleural effusion was also found for ibrutinib in a study evaluating the risk of pleural effusion with tyrosine kinase inhibitors (Mahé et al., 2018). Underlying mechanisms needs further investigation. Some authors have suggested that tyrosine kinase inhibitors could cause serosal inflammation (pleural and pericardial effusions) by their ability to inhibit multiple targets as Platelet Derived Growth Factor Receptor β (PDGFR-β) and Src family tyrosine kinases, which are involved in the maintenance of interstitial fluid tissue pressure and endothelial permeability (Kelly et al., 2009).

Our analysis highlights potential safety signals of ocular disorders: cataract in the two subgroups, uveitis, glaucoma and retinal ADRs (including retinal tear, vitreous detachment, retinal vascular occlusion) in patients aged <75 years. Since original studies on canine models have demonstrated corneal toxicity in animals receiving high doses of ibrutinib (US Food and Drug Administration, 2020), a concern emerged about the risk of cataract formation in ibrutinib-treated patients. Furthermore, blurred vision concerned 10% of ibrutinib-treated patients in the phase III RESONATE study (Byrd et al., 2014). Cataract was found to be one of the most commonly reported grade ≥3 adverse events in the 5-year follow-up of the phase III RESONATE study and was observed in 5.2% of patients (Burger et al., 2019). However, these results remain consistent with the background rate of cataract in the elderly (Asbell et al., 2005). Similarly, the higher reporting of glaucoma in the younger group may result from characteristics of ibrutinib-treated patients.

To date, ibrutinib-induced uveitis has been reported in only 3 case-reports (Arepalli et al., 2021; Bohn et al., 2021; Mehraban Far et al., 2021), in contrast with the 119 ICSRs documented in VigiBase. Selective pressure for Th1-mediated immunity has been proposed as a mechanism for ibrutinib-associated uveitis (Mehraban Far et al., 2021). Concerning retinal disorders, only macular edema has been previously reported with ibrutinib (Saenz-de-Viteri and Cudrnak, 2018; Mirgh et al., 2020). It is noteworthy that ibrutinib can penetrate the blood-brain barrier in mantle cell lymphoma patients (Bernard et al., 2015) and therefore reach the retina by passing through the blood-retinal barrier. Like MEK inhibitors (Huillard et al., 2014), retinal pigment epithelium toxicities may result from inhibition of mitogen-activated protein (MAP) kinases or upregulation of aquaporins in the retinal pigment epithelium (Daruich et al., 2018).

Concomitantly with a well-known risk of fall (US Food and Drug Administration., 2020), ibrutinib was associated with higher reporting of fractures on ibrutinib in both age groups. Except for hip fracture, patterns of fractures differ between age groups. Spinal (including spinal compression), foot and stress fractures reporting was significantly increased in patients aged <75 years, while lower limb, upper limb and stress fractures significantly increased in the older group. A recent retrospective study found a higher risk of spinal fracture in ibrutinib-treated patients for CLL (Laroche et al., 2020). In contrast, some authors have suggested that ibrutinib could be a potential therapeutic agent for certain osteoclast-related diseases, such as osteoporosis and rheumatoid arthritis. Ibrutinib inhibits osteoclast differentiation and function *in vitro* by regulating the expression of osteoclast-associated genes (Shinohara et al., 2014). Moreover, in the same study, oral administration of ibrutinib was shown to protect against bone loss in a murine model of osteoporosis. It should be noted that confounders including B-cell malignancies, or risk factors of fractures (e.g., body mass index, prevalence of smoking, specific comorbidities, serum vitamin D levels, or use of medications) could also affect our findings. Since BTK plays a role in bone resorption and metabolism, our results suggest a need for further assessments on the potential occurrence of fractures and osteoporosis in patients treated with ibrutinib.

Regarding endocrine and metabolic disorders, the SDR of hyponatremia is concordant with long-term safety data from the phase 3 RESONATE-2 study, where hyponatremia was found to be one of the most common grade ≥3 adverse events. Tyrosine kinase inhibitors treatment has been associated with syndrome of inappropriate antidiuretic hormone secretion, especially in patients under BCR-ABL inhibitors (Liamis et al., 2016). However, underlying mechanisms for ibrutinib-induced hyponatremia did not emerge from literature.

Nearly a dozen tyrosine kinase inhibitors have been implicated in hypothyroidism and definitive associations are known for 5 (axitinib, imatinib, pazopanib, sorafenib, and sunitinib) (Kust et al., 2016). Only one case-report described increased thyroid-hormone requirements in a thyroidectomized 80-year-old woman while on ibrutinib treatment (Mazori and Skamagas, 2021). The levothyroxine dose required to preserve a normal TSH decreased after ibrutinib discontinuation, indicating that ibrutinib-induced hypothyroidism is reversible. The authors hypothesize that the patient’s hypothyroid state was caused by type 3 deiodinase induction, leading to increased conversion of thyroxine into reverse triiodothyronine.

We found that depression was more frequently reported with ibrutinib in both groups, in comparison with anticancer drugs. Only anxiety is listed in the US product information (US Food and Drug Administration, 2020). As underlined above, ibrutinib is able to pass through the blood-brain barrier, and could as a result induce psychiatrics ADRs, such as anxiety or depression.

Lastly, to the best of our knowledge, ototoxicity of ibrutinib has not been previously reported. Because of increased risk of infections (including otitis) and bleeding events (including hemotympanum) in patients treated with ibrutinib, our results should be interpreted with caution. Off-target effects of ibrutinib, in particular VEGF inhibition, may explain ibrutinib-associated deafness. Anti-VEGF agents may induce hearing loss by causing a reduction in local blood flow and/or microvascular thrombosis in the labyrinth (Dekeister et al., 2016; Cheng et al., 2019).

### Strengths and Limitations

This work has several important strengths. The present study used the world’s largest and most representative spontaneous reporting database (VigiBase), which currently includes more than 124 million ICSRs from >130 countries. Reports from VigiBase represent data in the context of real-world settings, which have not been investigated in clinical trials (Bégaud and Montastruc, 2019; Montastruc et al., 2019). It used a validated method (i.e., disproportionality analysis) which was found to be able to detect unknown or rare safety signals (Montastruc et al., 2019).

From a statistical point of view, the combined use of two complementary disproportionality measures (i.e., PRR and IC) provides the most accurate estimate for drug-SADR associations, especially for associations with few cases. Moreover, given the characteristics of ibrutinib-patients, this study was performed trying to limit confounding of age. Subgroup analyses (i.e. patients aged <75 years and ≥75 years) improve sensitivity and precision and are clearly beneficial over crude analyses in large databases (Seabroke et al., 2016; Sandberg et al., 2020).

Our study has some limitations. Underreporting is an intrinsic limitation to research performed using pharmacovigilance data. It mostly concerns less severe and/or expected adverse drug reactions (Lopez-Gonzalez et al., 2009; Alatawi and Hansen, 2017). Furthermore, González-Rubio et al. (2011) found that ADRs are more often reported for elderly patients. Therefore, the potential impact of under-reporting on this work appears to be low. The use of all anticancer drugs as the reference group should have minimized any potential indication bias, even if differences in cancer types and patient characteristics may account for the discrepancies found between the two age groups. ADR reporting comes from heterogeneous sources in VigiBase, thus raising the possibility of incomplete information for time to onset or comorbidities. Consequently, the impact of risk factors on potential safety signals cannot be ruled out. In addition, the volume of ICSRs for a specific drug may be different between countries according to its extent of use or time of registration.

## Conclusion

Using a large-scale pharmacovigilance database, we found clinically relevant potential safety signals in patients exposed to ibrutinib, mainly ischemic heart diseases, pericarditis, uveitis, retinal disorders and fractures. Owing to the mandatory limitations of this study, these results need further confirmation using population-based studies. However, all of these potential safety signals should be considered in patient care and in clinical trial designs.

## Data Availability

The datasets presented in this article are not readily available. The data that support the findings of this study are available from the UMC. Restrictions apply to the availability of these data, which were used under license for this study. Data are available from the authors with the permission of the UMC. Requests to access the datasets should be directed to marion.allouchery@univ-poitiers.

## Abbreviations

ATC, anatomical therapeutic chemical, BCR, b-cell receptor; BTK, bruton’s tyrosine kinase; CLL, chronic lymphocytic leukemia; EMA, european medicines agency; FDA, US food and drug administration; IC, information component; IC025, credibility interval; ICSR, individual case safety report; IQR, interquartile range; MAP, mitogen-activated protein; MedDRA, medical dictionary for regulatory activities; MCL, mantle cell lymphoma, PDGFR-β, platelet derived growth factor receptor β, PRR, proportional reporting ratio; PT, preferred term; rT3, reverse triiodothyronine; SADR, serious adverse drug reaction; SDR, signal of disproportionate reporting; SmPC, summary of product characteristics; T4, thyroxin; UMC, uppsala monitoring center; VEGF, vascular endothelium growth factor; VEGFR, vascular endothelium growth factor receptor; WHO, world health organization; WM, waldenström macroglobulinemia; 95% CI, 95% confidence interval.
